# Case Report: PTEN Mutation Induced by anti-PD-1 Therapy in Stage IV Lung Adenocarcinoma

**DOI:** 10.3389/fphar.2022.714408

**Published:** 2022-05-23

**Authors:** Junjie Teng, Kai Zhou, Dongxiao Lv, Changshun Wu, Hong Feng

**Affiliations:** ^1^ Cancer Center, Shandong Province Hospital Affiliated to Shandong First Medical University, Jinan, China; ^2^ Department of Radiotherapy, The Third Affiliated Hospital of Shandong First Medical University, Jinan, China; ^3^ Department of Surgery, Shandong Province Hospital Affiliated to Shandong First Medical University, Jinan, China

**Keywords:** ErbB2 mutation, afatinib, adenocarcinoma cell lung carcinoma, PTEN mutation, immunotherapy

## Abstract

Lung cancer is the most common solid tumor in the worldwide. Targeted therapy and immunotherapy are important treatment options in advanced non-small cell lung cancer (NSCLC). The association of PTEN mutation and tumor immunotherapy is less established for patients with NSCLC. We present the case of an Asian woman diagnosed with stage IV lung adenocarcinoma harboring an ERBB2 mutation. She received Nivolumab treatment when her disease progresses after previous chemotherapy and Afatinib treatment. However, the patient did not response to Nivolumab. PTEN mutation was detected by next-generation sequencing (NGS) after treatment with Nivolumab. PTEN, a secondary mutation, may be served as a biomarker of resistance to anti-PD-1 immunotherapy in lung adenocarcinoma. The relationship between PTEN mutation and immunotherapy is complex and needs further study.

## Introduction

Non-small cell lung cancer (NSCLC) is the most common type of lung cancer accounting for approximately 85% of all lung cancers (Torre et al., 2016).

Targeted therapy is the standard treatment for advanced NSCLC with driver genetic alterations. The common driver genes changes in lung adenocarcinoma include EGER, ALK, ROS1. For advanced NSCLC patients without driver genetic alterations, immune checkpoint inhibitor (ICI) therapy is an alternative treatment option. Currently, the cell programmed death receptor 1 (PD-1) and its ligand (PD-L1) inhibitors are approved by FDA for the treatment of metastatic NSCLC after failed platinum-containing chemotherapy. Based on the data from CheckMate 057 trial (Vokes et al., 2018), Nivolumab, an anti-PD-1 antibody, was recommended as a subsequent therapy for patients with metastatic nonsquamous NSCLC who have progressed on or after first-line chemotherapy by the 2019 National Comprehensive Cancer Network Clinical Practice Guidelines. Nivolumab improved the 5-year overall survival rate in patients with advanced NSCLC from less than 5–16% (Gettinger et al., 2018). However, treatment response is merely observed in 16.9–20% of patients (Brahmer et al., 2015; Wu et al., 2019). Therefore, it is very important to look for biomarkers to predict which patients are most likely to benefit from immunotherapy. In addition, Gene mutations can make tumor patients sensitive or resistant to immunotherapy and impact on clinical drug selection and treatment effect. Studies have shown that loss-of-function mutations in the PBRM1 gene were associated with response to anti-PD-1 monotherapy in patient with clear cell renal cell carcinoma (Miao et al., 2018). However, mutations in the PTEN, JAK1 or JAK2 genes were related to primary or acquired resistance to immunotherapy (Peng et al., 2016; Zaretsky et al., 2016; George et al., 2017; Parikh et al., 2018). In fact, gene changes can also emerge after ICIs therapy. Researchers have performed comprehensive genomic profiling on melanoma samples treated with Nivolumab therapy. They observed that novel sets of mutations on-therapy were gained in progressive disease (PD) or stable disease (SD) patients (Riaz et al., 2017).

PTEN is a known tumor suppressor whose mutation rate is only second to p53 in human tumors. PTEN facilitates fundamental anti-oncogenic tasks and its encoded PTEN protein can promote cell cycle arrest in G1 phase and apoptosis, thereby inhibiting tumor cell growth. PTEN can also inhibit cell invasion and metastasis (Kim et al., 2014). However, deletion or mutation of PTEN in NSCLC are founded only inapproximately 2–7% of cases. Researchers founded that EGFR-mutant patients having PTEN deletion showed a more dismal PFS and OS than those with intact PTEN (Wang et al., 2019). Zhao and others performed a longitudinal study of 66 glioblastomas patients, They founded that PTEN mutations significantly enrich in non-responders (Zhao et al., 2019). Reports on the relationship between PTEN mutations and immunotherapy in NSCLC are very rare. Herein, we present the case of a patient who had no PTEN mutation found prior to Nivolumab therapy, but developed PTEN mutation after 2 months of Nivolumab treatment.

## Case Presentation

**FIGURE1:**
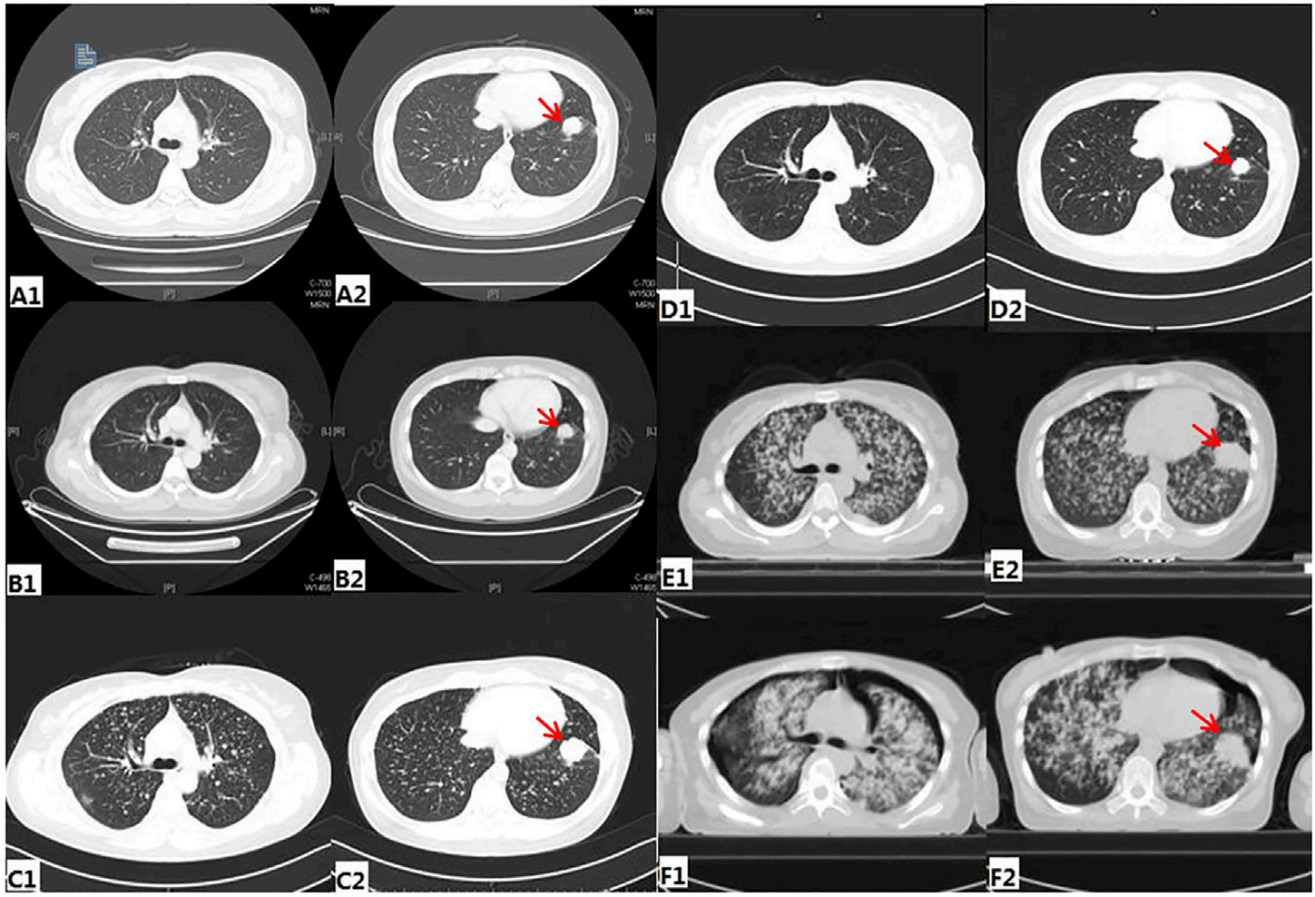
Radiologic findings during treatment **(A)** before any treatment **(B)** Post-treatment image after three cycles of pemetrexed and cisplatin **(C)** before Afatinib treatment **(D)** CT of the chest showing PR after 1month of Afatinib **(E)** before nivolumab treatment **(F)** CT of the chest showing PD after 4 cycles of Nivolumab.

A 40-year-old Chinese woman who had never smoked presented to our center due to right shoulder pain in September 2016. Physical examination revealed an enlarged lymph node about 1 × 1 cm in size placed on the left supraclavicular fossa. A chest computed tomography (CT) scan indicated a space-occupying lesion in the left lower lobe with miliary distribution of multiple tiny nodules in both lungs, and local bone destruction at the right scapula (Figure 1A). Biopsy of left supraclavicular fossa lymph node showed metastatic adenocarcinoma. She was diagnosed with stage IV lung adenocarcinoma with metastases. Amplification Refractory Mutation System PCR (ARMS PCR) was used to measure the tissue biopsy for four common mutations of EGFR exon 18–21, and it showed no mutation was found (Table 1).

**TABLE 1:** List of four gene test results during the whole treatment of patient.

Time	Discovery Technique	Human Material	Detection Range	Gene Mutations	Mutation Frequency (%)		
					Tissue	Plasma	Pleural Effusion
September 2016	ARMS PCR	Tissue	EGFR exon 18–21	None	—	—	—
March 2017	NGS	Tissue/Plasma	123 Gene	ERBB2 (P.V777delins VGSP)	14.1	28.8	—
				TP53 (p.Y126N)	9.8	30.8	—
March 2018	NGS	Tissue/Plasma	139 Gene	BRAF (p.M279L)	—	1.7	—
				ERBB2 (P.V777delins VGSP)	39.7	39.9	—
				TP53 (p.Y126N)	37.0	25.3	—
September 2018	NGS	Plasma/Pleural Effusion	139 Gene	PTEN (c.A1027-2T)	—	—	3.5
				ERBB2 (P. VGSP)	—	55.8	85.1
				TP53 (p.Y126N)	—	21.1	67.0

ARMS PCR, amplification refractory mutation system PCR; NGS, next generation sequencing.

The patient received six cycles of Pemetrexed and Cisplatin which result in partial response (PR) (Figure 1B). Then she received three cycles of Pemetrexed monotherapy for the next 2 months, during which the scapula metastases were treated with radiotherapy. Thereafter, progression of lung was demonstrated in CT scan in May 2017 (Figure 1C). So, she underwent second-line chemotherapy. After two cycles of chemotherapy with Gemcitabine combined with lobaplatin, multiple intracranial metastasis was observed on brain magnetic resonance imaging. In order to determine whether she can benefit from targeted therapy, 123 gene exons were detected by NGS with samples ofpatients’ blood and previously punctured lymph node tissues. A mutation frequency of 28.8 and 14.1% exon 20 ERBB2 insertion mutations were respectively found. Besides, a TP53 mutation was further detected (Table 1). In June 2017, she startedAfatinib 40 mg daily. Encouragingly, CT imaging showed radiological PR in bilateral lung lesions after 1 month (Figure 1D). Until August 2017, though the tumor burden in the lung had maintained stable state, the fifth lumbar vertebra and iliac metastases were observed by CT scan. She continued to receive monotherapy with Afatinib for the next 6 months with slow progression. Meanwhile, the patient received radiotherapy for lumbar vertebra and iliac metastases. However, Afatinib treatment was interrupted after a total of 11 months therapy due to rapid progression of pulmonary lesions and brain metastases (Figure 1E). In March 2018, another biopsy was performed at supraclavicular metastatic lymph node. Compared with the last testing consequence, the frequency of TP53 mutation decreased, and the frequency of ERBB2 insertion mutation increased in biopsy plasma. Otherwise, a BRAF mutation was detected only in plasma at a frequency of 1.7% (Table 1).

**FIGURE 2:**
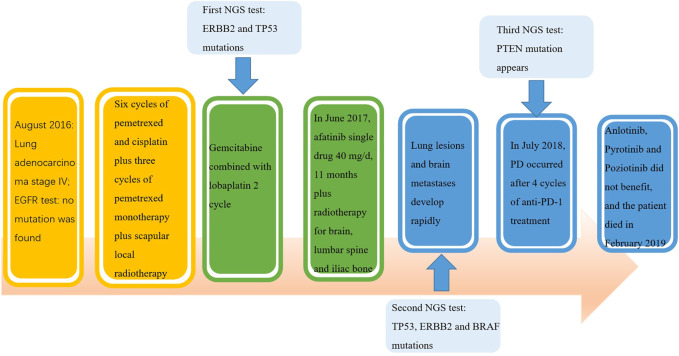
The whole course of treatment of the patient.

In July 2018, the patient has been enrolled in a clinical trial to receive anti-PD-1 treatment with the 240 mg q2w regimen of Nivolumab. Unfortunately, she does not benefit from immunotherapy. After 4 cycles of Nivolumab, she developed further PD in the lung. Compared with the CT images before immunotherapy, it appeared pleural effusion (Figure 1F). At this time, patient’s pleural effusion was taken as samples for the third gene detection by NGS. Compared with the second test result, it can be seen that PTEN mutation appeared, TP53 mutation frequency decreased, and ERBB2 gene insertion mutation frequency continued to increase (Table 1). However, BRAF gene mutation was not detected. Tumor mutation burden (TMB)of the patient was 6.7 mut/mb. She was subsequently treated with Anlotinib, Pyrotinib and Poziotinib successively. Unfortunately, she did not profit from the treatment of these drugs. The patient event-ually died in February 2019 after 6 months immunotherapy. The entire treatment process of the patient is shown in Figure 2.

## Discussion

This report of an advanced NSCLC patient who has received comprehensive treatment, including chemotherapy, radiotherapy, targeted therapy and immunotherapy. Checkpoint inhibitors can activate the immune system, enhance anti-tumor activity and prolong the survival of patients with NSCLC (Parry et al., 2005). During the treatment of this case of NSCLC, we dynamically monitor the genetic alterations of the tumor and found that PTEN mutation appeared after Nivolumab treatment resistance.

PTEN is an antitumor gene, and its inactivation mutation can lead to continuous activation of mTOR pathway, so that tumor growth cannot be controlled (Kim et al., 2014). Previous studies have reported that NSCLC patients who were treated with Nivolumabcombine with ipilimumab could not benefit from immunotherapy if PTEN mutation occurred in pretreatment tissues (Hellmann et al., 2018). Parikh, A.R reported a patient with NSCLC who had PTEN and STK11 gene mutations. Although the PD-L1 positive rate of the tumor cells reached 80%, neither Nivolumab nor Pembrolizumab achieved sustainable long-term efficacy (Parikh et al., 2018). George and his colleagues identified a metastatic uterine leiomyosarcoma patient who has experienced complete tumor remission for >2 years on pembrolizumab monotherapy. They analyzed the sole treatment-resistant metastasis to explore mechanisms of immunotherapy resistance, and found that the treatment-resistant tumor uniquely harbored biallelic PTEN loss genetically (George et al., 2017). All the above reports suggest that PTEN deletion or mutation in tumors may promote resistance to the antitumor immune response. To the best of our knowledge, we have shown for the first time that PTEN mutation appeared after treatment with anti-PD-1 (Nivolumab) therapy in NSCLC. Further indicates that secondary mutation in PTEN may lead to immunotherapy resistance. The prediction and evaluation of the efficacy of ICIs are very complicated. Genomic alterations that may occur before or after immunotherapy have essential effects on the efficacy of immunotherapy (Riaz et al., 2017). Chromosome instability in cancer drives phenotypic adaptation during tumor evolution (Sansregret et al., 2018). The theory of clonal evolution describes tumor genesis as a process of mutation acquisition. PTEN mutation in the patient we report may be the outcome of tumor’s clonal evolution (Mohammad et al., 2014; Peng et al., 2016). It has been s-uggested that genomic instability is related to the subclonal evolution of tumor cells. Genomic instability includes genomic duplication, loss and amplified gene point mut-ations. This mode enables tumor cells to generate more growth-adapted subpopulatio-ns and select favorable subclonal amplification to obtain mutations (Hiley et al., 2014).

Loss of PTEN can activate the PI3K pathway, leading to decreased responserates to immunotherapy (Rizvi and Chan, 2016). Whether a PI3K inhibitor could synergize with immunotherapy treatments? In PENG’s study, a genetically engineered murine model that spontaneously developed BRAF-mutant, PTEN-null melanomas were randomly treated with PI3Kβ inhibitor, anti–PD-1 antibody, or combination of PI3Kβ inhibitor and anti–PD-1. The results showed that the combination of the two drugs significantly improved tumor growth inhibition and survival of the mice. Inhibition of PI3K pathway can improve the efficiency of immunotherapy (Peng et al., 2016). This study enlightens us that the combination of ICIs and other drugs may enhance the efficacy of immunotherapy and improve patient survival in the future.

Notably, genetic alterations in this patient‘s cancer included not only PTEN mutation, but also ERBB2 and TP53 mutations. Then, what are the influence of ERBB2 or TP53mutations on immunotherapy? BT Li et al. reported seven patients with HER2 mutant lung adenocarcinomas who received anti-PD1 immune checkpoint inhibitors, and none responded (Li et al., 2018). Recent studies showed that EGFR mutations, ALK rearrangements and MET exon 14 mutants are linked to low response rates to PD-1 pathway blockade in NSCLC (Gainor et al., 2016; Sabari et al., 2017). Response rates of immunotherapy may be similarly low in patients with HER2 mutations. In addition, Among clinical drugs for lung cancer recommended by FDA/NCCN guidelines, mutations in exon 20 of ERBB2 (HER2) gene can cause resistance to first-generation EGFR-Tkis, but respond to second-generation irreversible EGFR-Tkis, such as afatinib drugs. Prior studies have manifested that TP53 mutation significantly increased expression of immune checkpoints and activated T-effector and interferon-γ signature. This change in tumor microenvironment indicates a better immune response to tumor cells. Lung adenocarcinoma patients with TP53 mutation, especially those with co-occurring TP53/KRAS mutations, showed remarkable clinical benefit to PD-1 inhibitors (Dong et al., 2017). Another study also demonstrates that TP53 mutations were associated with response and longer survival under ICIs in advanced NSCLC (Assoun et al., 2019). Unfortunately, although TP53 mutation exists in the patient we reported, she does not response to Nivolumab.

## Conclusion

In summary, the case we reported indicate that the effect of immunotherapy is related to genetic abnormality. PTEN mutation may be used as biomarker to predict resistance to anti-PD1 immune checkpoint inhibitors in lung adenocarcinoma. However, larger samples are required for validation. There is still a long way to go to understand the role and regulatory mechanism of PTEN in immunology. It is hope that in-depth research on these aspects in the future will contribute to the development of PTEN-related therapies for human cancer. The exploration of combination therapy in patients with drug resistance has the potential to improve the response rate of NSCLC with PTEN mutation to anti-PD-1 treatment. Afatinib, as a non-reversible tyrosine kinase inhibitor of HER2, is a potential treatment option for patients with advanced lung adenocarcinoma with ERBB2 20 exon insertion. In addition, repeated NGS testing in the process of tumor treatment has not only guided targeted therapy, but also contributed to determine the efficacy of immunotherapy and understand the resistance mechanism of immunotherapy. NGS detection can track find more genetic mutations, but now the high cost, less cost, we repeat biopsy in the treatment process, blood samples at differ-ent times, puncture, the results of multiple genes, tracking guidance significance. In a-ddition, patients with advanced lung adenocarcinoma patients, early attention to treat-ment effect, respect the wishes of the patients with late treatment, in order to improve the quality of life, Prolonging life is the main purpose.

## Data Availability

The original contributions presented in the study are included in the article/Supplementary Material, further inquiries can be directed to the corresponding authors.
