# Erratum: Case report: PTEN mutation induced by anti-PD-1 therapy in stage IV lung adenocarcinoma

**DOI:** 10.3389/fphar.2022.1058178

**Published:** 2022-10-20

**Authors:** Frontiers Production Office

**Keywords:** ErbB2 mutation, afatinib, adenocarcinoma cell lung carcinoma, PTEN mutation, immunotherapy

Due to a production error, the author affiliations were listed incorrectly, as “Junjie Teng^1,2,†^, Kai Zhou^2,†^, Dongxiao Lv^2^, Changshun Wu^3,^* and Hong Feng^2,^*” instead of “Junjie Teng^1,2,†^, Kai Zhou^1,†^, Dongxiao Lv^1^, Changshun Wu^3,^* and Hong Feng^1,^*”.

The publisher apologizes for this mistake. The original version of this article has been updated.

